# The impact of moral judgment on bystanders’ interpersonal trust: the mediating role of trustworthiness

**DOI:** 10.3389/fpsyg.2024.1440768

**Published:** 2025-01-03

**Authors:** Zhen Zhang, Xia Cai, Weiwei Gao, Zengtong Zhang, Chunhui Qi

**Affiliations:** ^1^Faculty of Education, Henan Normal University, Xinxiang, China; ^2^Faculty of Education, Henan University, Kaifeng, China; ^3^Hongqi District Second Experimental Primary School, Xinxiang, China

**Keywords:** interpersonal trust, moral judgment, trustworthiness, mediation effect, adolescent

## Abstract

Interpersonal trust is the premise and foundation of encouraging cooperation in this age of rapid progress. The purpose of this study was to investigate how moral judgment affects bystanders’ interpersonal trust and its internal mechanisms when there are ethical transgressions. The moral judgment of the evaluators was divided into three categories—opposition, neutrality and approval—on the basis of the moral transgressions of the offenders. Three moral judgment circumstances were randomly assigned to 143 primary school pupils, and the assessors scored the children via trustworthiness and trust scales. According to the findings, interpersonal trust is significantly predicted by moral judgment. Compared with neutral judgment, opposing moral violations significantly improves bystanders’ interpersonal trust in the evaluator, whereas approving moral violations does not significantly predict interpersonal trust. Trustworthiness plays a mediating role in the influence of moral judgment on interpersonal trust. Compared with neutral judgment, trustworthiness mediates the influence of opposed judgment on interpersonal trust rather than the influence of approved judgment on interpersonal trust. The findings demonstrate that moral opposition to transgressions influences interpersonal trust either directly or indirectly through trustworthiness.

## 1 Introduction

Interpersonal trust is the cornerstone of human cooperation and collaboration, permeating all aspects of social life. As a lubricant for social interaction, trust not only plays a crucial role in initiating, establishing, and maintaining intimate relationships, but also contributes to the prosperity of groups, organizations, and nations (Dunning et al., 2019; Weiss et al., 2021). Over the past two decades, the evolution and impact factors of trust behaviors in humans and other populations have become a central topic of discussion in fields such as economics (Chetty et al., 2021), politics (Carlin et al., 2022), psychology (Weiss et al., 2021), and cognitive neuroscience (Krueger and Meyer-Lindenberg, 2019), and have been extensively explored. By means of economic game tasks and self-report questionnaires, a large number of studies have discovered that trusting others is a ubiquitous social preference (Dunning et al., 2014), possesses a certain degree of biological heritability (Riedl and Javor, 2012), and is prone to the interactive influence of personality and situational factors (Weinschenk and Dawes, 2019).

In the context of interpersonal interactions, individuals frequently depend on a range of social cues—such as facial characteristics, reputation information, and observable behaviors—to assess the trustworthiness of others and ultimately determine whether to extend their trust. Costly signaling theory posits that expensive social behaviors, like third-party interventions, indicate trustworthiness to bystanders, influencing their interpersonal trust toward to the interveners (BliegeBird and Smith, 2005; Gintis et al., 2001). Third-party punishers are viewed as more trustworthy in economic game tasks than persons who do not carry out punishment, according to Jordan et al. (2016). Other studies have found that corrupt third parties undermine trust and prosocial behavior between people (Spadaro et al., 2023). Sun et al. (2023) also found that third-party punishment affected bystanders’trust in punishers in both the in-group and out-group conditions. In addition, studies have analyzed the process by which punishment systems shape trust (Olcina and Calabuig, 2021). However, there are not many work exploring the impact of non-third party interventions on interpersonal trust. Moral judgment is a way of non-third-party intervention, and will it have a similar impact on interpersonal trust?

Moral judgment is a crucial way for individuals to intervene in moral transgressions (Lergetporer et al., 2014). As a type of social cue, moral judgment can convey information such as the trustworthiness of the person who is assigning judgment (Connelly et al., 2011; Haidt, 2001). Nonetheless, there are certain limitations in earlier studies on moral judgment and interpersonal trust. First, previous studies have discussed separately two types of moral judgment, disapproval and approval, but few studies have examined and compared the two together (Bostyn et al., 2023; Everett et al., 2016); however, simultaneously exploring the relationship between the two and interpersonal trust plays an important role in motivating and strengthening human cooperation (Guglielmo and Malle, 2019). Second, the behavioral game task—which is less common in everyday life—was applied in the majority of earlier studies (Karlan, 2005; Thielmann and Hilbig, 2015), thus limiting the generalizability of the findings to real-world contexts. Third, while earlier research has been conducted on adult populations, it is crucial to concentrate on adolescents to understand how the relationship between moral judgment and interpersonal trust develops because adults and adolescents have different age features (Bostyn and Roets, 2017; Everett et al., 2021; Towner et al., 2023). Adolescence is an important period for the development of individual moral cognition. According to the Kohlberg’s stages of moral development, students in this period were in the stage of seeking directional recognition, and their moral values were oriented by interpersonal harmony (Walker, 1982). The study of the relationship between the moral judgment and the interpersonal trust during this period can help them to establish a good peer relationship and promote the cooperation and development of the future society. Moreover, previous studies mostly focus on adult groups, and the socialization degree of adult groups is much higher than that of adolescent (Simpson et al., 2013; Bostyn and Roets, 2017; Bostyn et al., 2023; Everett et al., 2016). The study of adolescent is helpful to clarify the development process of moral judgment affecting the interpersonal trust of bystanders and enrich the content of this field.

### 1.1 Moral judgment and interpersonal trust

Human society is constrained by various moral norms. Social moral norms can be effectively upheld, and social justice and fairness can be promoted by individual intervention in moral transgressions. The term “moral judgment” primarily refers to the perceiver’s assessment of a breach of moral standards, and it may be classified into four types: evaluation judgment, normative judgment, moral error judgment, and blame judgment (Malle, 2021). According to costly signaling theory, opposing moral violations can send a signal to others that people may trust the evaluator more in cases of risk and uncertainty (BliegeBird and Smith, 2005). Approving moral transgressions is contrary to modern society’s norms, but it can also send a message to others that the evaluator is not someone to trust on a personal level (Gintis et al., 2001). Moreover, it has been demonstrated that a person’s moral judgment of immoral activity increases his or her degree of trust (Simpson et al., 2013). Kennedy and Schweitzer (2018) also reported that when accusing others of immoral behavior, the individual sends a signal of his or her moral character, thus increasing the other person’s interpersonal trust. Other studies have shown that a person’s approved judgment of immoral activity can negatively affect the perception of bystanders and reduce their level of trust is that individual (Uhlmann et al., 2013; Bostyn and Roets, 2017). Therefore, Hypothesis 1 is as follows: Interpersonal trust is impacted by moral judgment. Compared with neutral judgment, opposing moral violations positively predicts interpersonal trust, and approving moral violations negatively predicts interpersonal trust.

### 1.2 Trustworthiness as a potential mediator

Trustworthiness is the perception of qualities such as individual ability, benevolence, and integrity (Colquitt et al., 2007; Mayer et al., 1995). On the one hand, studies have indicated that observers deduce an individual’s personality from the moral judgments made by others (Kreps and Monin, 2014; Sacco et al., 2017; Uhlmann et al., 2013). The costly signaling theory also holds that an individual’s judgment of immoral behavior sends a signal that indicates the individual’s ability, benevolence and integrity, which constitute the perception of the individual’s trustworthiness (Gintis et al., 2001; Mayer et al., 1995). In other words, moral judgment affects trustworthiness. Research has demonstrated that moral judgment has an impact on trustworthiness and that people tend to view strong moral judges as more trustworthy than weak moral judges (Simpson et al., 2013). According to Everett et al. (2016), moral judgment can be used to infer an individual’s trustworthiness. Therefore, moral judgment can play a role in establishing trustworthiness.

On the other hand, according to the ABI model proposed by Mayer et al. (1995), trust can be examined from three perspectives—ability, benevolence and integrity—in which ability and integrity contribute to cognition-based trust (McAllister, 1995) whereas benevolence contributes to emotion-based trust (Dirks and Ferrin, 2002). Some studies have also shown that characteristics such as perceived individual ability, benevolence and integrity have a direct effect on the degree to which others trust a person (Sun et al., 2023; Wang and Murnighan, 2017). All of these studies highlight the importance of trustworthiness in building interpersonal trust. Kennedy and Schweitzer (2018) also reported that the perception of integrity plays a mediating role between alleging unethical behavior and interpersonal trust. Therefore, Hypothesis 2 is as follows: Trustworthiness plays a mediating role in the influence of moral judgment on interpersonal trust. Compared with neutral people, opposing moral violations increases interpersonal trust by increasing the perception of trustworthiness, and approving moral violations reduces interpersonal trust by reducing that perception.

To test the above hypothesis, this study improved upon previous studies by using adolescents as the subjects and proposed the use of a school class moral violation scenario to explore the influence of moral judgment on interpersonal trust and the mediating role of trustworthiness. This study largely uses a paper experiment with a single-factor, three-level interexperimental design to investigate the relationships among moral judgment, trustworthiness, and interpersonal trust. The three moral judgments are opposed, neutral, and approved.

## 2 Method

### 2.1 Participants

A total of 162 questionnaires were collected from a primary school in Henan Province, among which 19 questionnaires were excluded because of missing items. Ultimately, 143 questionnaires were valid, and the effective response rate of the questionnaires was 88.27%. Our sample size was determined through an *a priori* power analysis, assuming an *α* of 0.05 and a power of 0.80, indicating that the minimum effect size we had power to detect was a medium effect of *f* = 0.25 (Faul et al., 2007). The subjects’ ages ranged from 11 to 13 years, with an average age of 11.29 years (*SD* = 0.50). Of the 143 subjects, 69 (48.30%) were female, and 74 (51.70%) were male. All the subjects were physically and mentally healthy, had no history of mental illness, were right-handed, and had normal or corrected-to-normal vision. Ethics committee approval was obtained from the Faculty of Education at Henan Normal University, and protocol adherence to the Declaration of Helsinki was ensured.

### 2.2 Experimental materials

#### 2.2.1 Moral violation scenario

The moral violation scenario employed in this study was adapted from Dhaliwal et al. (2020). “Imagine this scene: classmate A was loud in front of all classmates and teachers during a class meeting last week.” This was the exact description of the class violation used in this study. The three types of moral judgments were as follows: approved (Monitor B praises classmate A and believes it is morally appropriate), neutral (Monitor B does not condemn or praise classmate A), and opposed (Monitor B condemns classmate A and considers it morally inappropriate). Four students were asked to participate in a pretest to ensure that the subjects grasped the material and the situation before the official test. After the experiment, the subjects were interviewed, and all the subjects correctly understood the situation and related questions.

#### 2.2.2 Trustworthiness scale

This scale is adapted from the trustworthiness scale proposed by Mayer and Davis (1999). The scale is divided into three dimensions—ability, integrity and benevolence—with a total of 17 items. We changed “top management” in the original scale to “monitor B” and “work” to “class work.” All other parts remained unchanged. Questions 1–6 measure ability (e.g., “Monitor B is very capable of performing class work”); questions 7–11 measure benevolence (e.g., “My needs and wishes are very important to monitor B”); and questions 12–17 measure integrity (e.g., “Monitor B has a strong sense of justice”). The scale was rated on a 5-point Likert scale, where 1 represented complete disagreement and 5 represented complete agreement. Higher scores on the scale denoted greater trustworthiness. The internal consistency coefficient of this scale in this study was 0.92, and the internal consistency coefficients of the ability, benevolence and integrity dimensions were 0.90, 0.81, and 0.73, respectively. We performed a confirmatory factor analysis of this scale, and the results are as follows: χ^2^ = 190.73, *df* = 114, χ^2^/*df* = 1.67 (*p* < 0.001), TLI = 0.93, CFI = 0.94, RMSEA = 0.07, and SRMR = 0.05.

#### 2.2.3 Trust scale

The trust scale used in the Ng and Chua study contains 8 items, in which questions 1--4 measure cognitive trust and questions 5–8 measure emotional trust (Ng and Chua, 2006). We have adapted this scale. We changed “they” in the original scale to “monitor B” and changed “teamwork” to “class work.” A representative item for measuring cognitive trust is “Monitor B is the person who takes class work seriously.” A representative item for measuring emotional trust is “You can freely talk to monitor B about your difficulties in learning and know that monitor B is willing to listen.” The scale was rated on a 5-point Likert scale, where 1 represented complete disagreement and 5 represented complete agreement. The internal consistency coefficient of this scale in this study was 0.88, and the internal consistency coefficients of the cognitive trust and emotional trust subscales were 0.91 and 0.80, respectively. We performed a confirmatory factor analysis of this scale, and the results are as follows: χ^2^ = 19.62, *df* = 11, χ^2^/*df* = 1.78 (*p* < 0.001), TLI = 0.97, CFI = 0.99, RMSEA = 0.07, and SRMR = 0.04.

## 3 Results

### 3.1 Descriptive statistics and correlation analysis

Table 1 displays the descriptive statistics and correlation analysis results for moral judgment, trustworthiness and interpersonal trust. The results of the correlation analysis reveal that moral judgment was significantly negatively correlated with both trustworthiness and interpersonal trust and that trustworthiness was significantly positively correlated with interpersonal trust.

**Table 1:** Descriptive statistics and correlation analysis results (*N* = 143).

	*M* (*SD*)	1	2	3
1 Moral judgment	2.03 (0.80)	1		
2 Trustworthiness	2.91 (0.94)	−0.54***	1	
3 Interpersonal trust	2.91 (1.08)	−0.55***	0.87***	1

Moral judgment: Opposed judgment = 1, Neutral judgment = 2, Approved judgment = 3. ***p < 0.001.

### 3.2 Preliminary analyses

**Figure 1:**
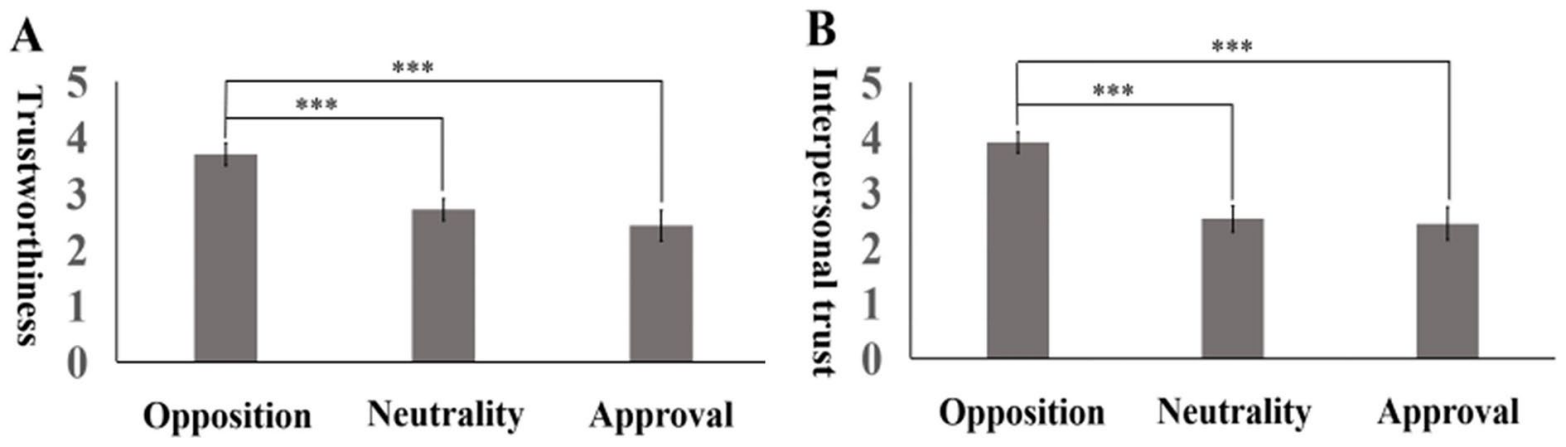
Differences in the effects of moral judgment on trustworthiness **(A)** and interpersonal trust **(B)**. ***p < 0.001.

One-way ANOVA was conducted with moral judgment as the independent variable and trustworthiness and interpersonal trust as the dependent variables. The results are shown in Figure 1. When trustworthiness was used as the dependent variable, the main effect of moral judgment was significant, *F* = 32.99, *p* < 0.001. Multiple comparisons reveal that the effect in the opposed group (*M* = 3.70, *SD* = 0.65) was significantly greater than that in the neutral group (*M* = 2.71, *SD* = 0.70) and the approved group (*M* = 2.43, *SD* = 0.95), whereas there was no significant difference between the neutral and approved groups. When interpersonal trust was used as the dependent variable, the main effect of moral judgment was significant (*F* = 44.99, *p* < 0.001). Multiple comparisons reveal that the effect in the opposed group (*M* = 3.94, *SD* = 0.62) was significantly greater than that in the neutral group (*M* = 2.53, *SD* = 0.87) and the approved group (*M* = 2.41, *SD* = 1.00), whereas there was no significant difference between the neutral and approved groups.

### 3.3 Mediation model

The mediating effect of trustworthiness between moral judgment and interpersonal trust was examined via SPSS 25.0 and Mplus 8.3. Moral judgment is dummy coded prior to the mediation effect analysis because it is a three-categorical variable. The neutral judgment group was set as the reference group to further reveal the causal relationship between moral judgment and interpersonal trust by comparison with the opposed judgment group and the approved judgment group. The dependent variable was interpersonal trust. All variables were normalized prior to the examination of the mediating effect. The results of the regression analysis are presented in Table 2.

**Table 2:** Regression analysis of moral judgment, trustworthiness and interpersonal trust.

Regression equation		Overall fit index			Significance of the regression coefficient	
Outcome variable	Predictor variable	*R*	*R* ^2^	*F*	*β*	*t*
Interpersonal trust	Opposed judgment (vs. neutral judgment)	0.63	0.39	44.99***	0.60	8.06***
	Approved judgment (vs. neutral judgment)				−0.05	−0.67
Trustworthiness	Opposed judgment (vs. neutral judgment)	0.57	0.32	32.99***	0.48	6.14***
	Approved judgment (vs. neutral judgment)				−0.15	−1.84
Interpersonal trust	Opposed judgment (vs. neutral judgment)	0.89	0.79	169.57***	0.23	4.64***
	Approved judgment (vs. neutral judgment)				0.06	1.34
	Trustworthiness				0.76	15.98***

All variables in the model are brought back into the equation after standardized processing. ***p < 0.001.

According to the regression coefficient of the regression equation with moral judgment as the predictor variable, interpersonal trust as the outcome variable and the results of the significance test, opposed judgment (vs. neutral judgment) can significantly positively predict interpersonal trust (*β* = 0.60, *p* < 0.001), but approved judgment (vs. neutral judgment) cannot significantly predict interpersonal trust. According to the regression coefficient of the regression equation with moral judgment as the predictor variable, trustworthiness as the outcome variable and the results of the significance test, opposed judgment (vs. neutral judgment) can significantly positively predict trustworthiness (*β* = 0.48, *p* < 0.001), but approved judgment (vs. neutral judgment) cannot significantly predict trustworthiness. The regression coefficient and significance test results show that when moral judgment and trustworthiness both affect interpersonal trust, opposed judgment (vs. neutral judgment) can significantly positively predict interpersonal trust (*β* = 0.23, *p* < 0.001), approved judgment (vs. neutral judgment) cannot significantly predict interpersonal trust, and trustworthiness can significantly positively predict interpersonal trust (*β* = 0.76, *p* < 0.001).

**Figure 2:**
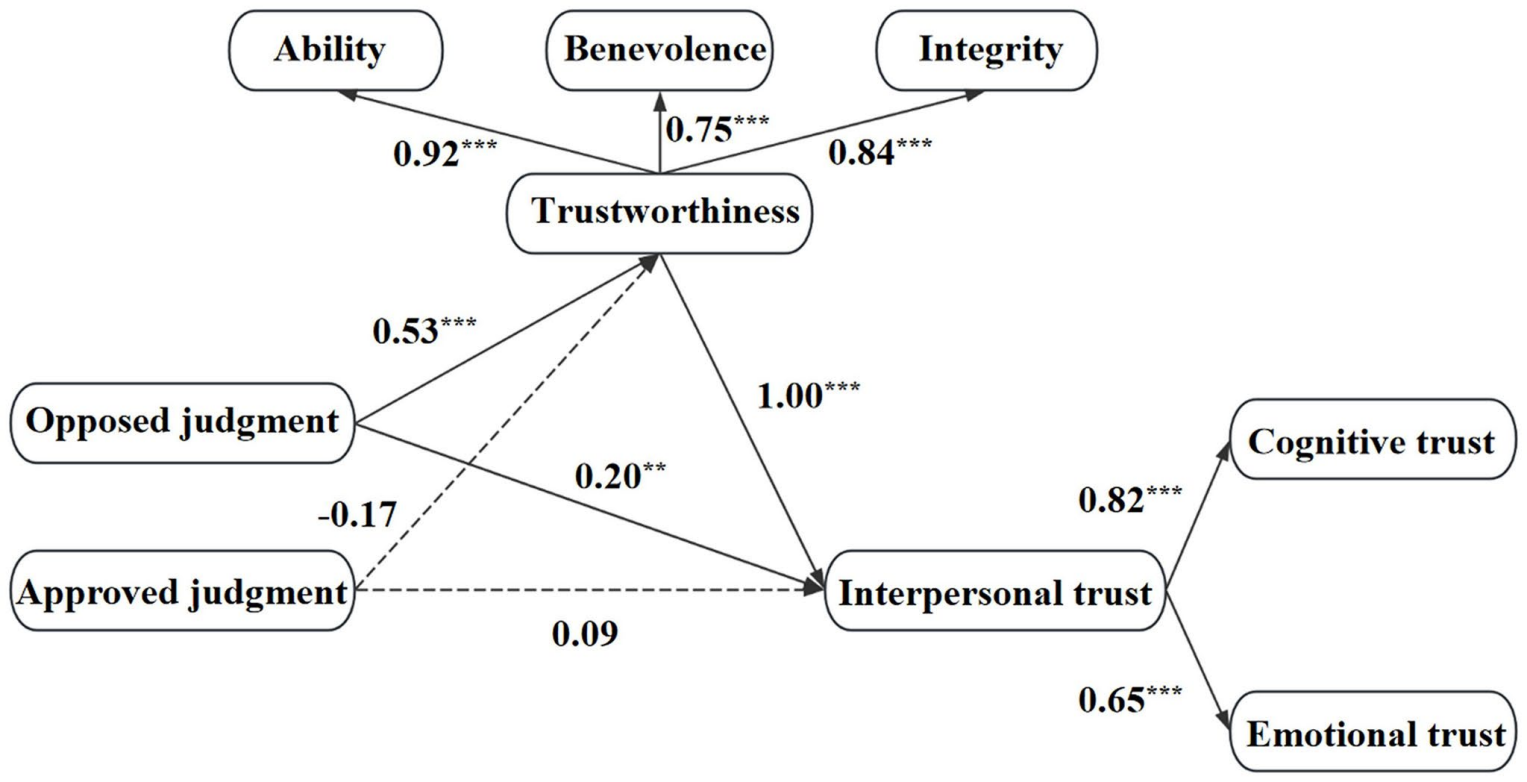
Mediating effect diagram of trustworthiness. ***p < 0.001.

This study tested the mediating effect of trustworthiness via a structural equation model to further identify the mediating role. The fit indices of the structural equation model are represented by χ^2^, CFI, TLI, RMSEA and SRMR. On the basis of this analysis, the results indicate that while the model fit is acceptable given the study’s context, it is not ideal, with χ^2^ = 54.22, *df* = 18, χ^2^/*df* = 3.01 (*p* < 0.001), TLI = 0.88, CFI = 0.94, RMSEA = 0.12, and SRMR = 0.06. The two latent variables are trustworthiness, i.e., ability, benevolence and integrity; and interpersonal trust, i.e., cognitive trust and emotional trust, with the neutral judgment group serving as the reference variable and the independent variables being coded as virtual variables. With trustworthiness serving as the mediating variable and interpersonal trust serving as the dependent variable, the results are shown in Figure 2. The analysis with trustworthiness serving as the mediating variable reveals that the total effect of the opposed judgment group was significant (c_1_ = 0.77, *p* < 0.001), the direct effect was significant (c’_1_ = 0.20, *p* < 0.05), and the indirect effect through trustworthiness was also significant (ab_1_ = 0.57, 95% CI [0.41, 0.77]). However, the total effect, direct effect, and indirect effect through trustworthiness of the approved judgment group were not significant.

## 4 Discussion

This study indicates that moral judgment can influence bystanders’ perceptions of trustworthiness and interpersonal trust. This is in line with previous studies (Simpson et al., 2013). For trustworthiness and interpersonal trust, this study revealed that different moral judgments can lead to different levels of trustworthiness perceptions and interpersonal trust. Specifically, the perception of trustworthiness and interpersonal trust of evaluators who make opposed judgments is greater than that of evaluators who make neutral judgments and approved judgments, whereas there is no significant difference in the perceived trustworthiness and interpersonal trust levels between evaluators who make neutral judgments and those who make approved judgments.

Opposition to violators in the face of moral transgressions highlight the values and positions of the evaluator. People are more likely to positively identify with an evaluator and view them as trustworthy when they believe that the evaluator shares their same values or the recognized values of their social group (Connelly et al., 2011; Haidt, 2001). This phenomenon also increases people’s interpersonal trust. Moreover, costly signaling theory suggests that opposing moral violations sends a signal to bystanders that the evaluator is trustworthy, thereby suggesting that the evaluator is more likely to be trusted in cases of risk and uncertainty (BliegeBird and Smith, 2005; Gintis et al., 2001). Indirect reciprocity theory also states that opposed judgments emphasize that moral violations are incorrect, prevent violators from harming others, safely safeguard the interests of others, maintain fairness and order within the group, and conclude that people are more likely to form positive cognitions of such assessors (Nowak and Sigmund, 2005). Therefore, people will trust evaluators who make opposed judgments more than those who make neutral and approved judgments. Moreover, it is possible that the study’s relatively minor moral violations have less severe consequences, so there is no significant difference in the perceived trustworthiness and interpersonal trust levels between the evaluators who make neutral judgments and those who make approved judgments.

In this study, trustworthiness plays a partial mediating role in the effect of moral judgment on interpersonal trust. That is, moral judgment affects the interpersonal trust of bystanders by influencing their perception of trustworthiness. A finding that is consistent with previous findings (Kennedy and Schweitzer, 2018; Jordan et al., 2016; Simpson et al., 2013). Opposed judgments can prevent such behavior in the future, safely safeguard the interests of others, maintain fairness and order within the group, and highlight the individual’s own moral standards by indicating the individual’s attitude toward moral violations. Opposed judgment also reveals the individual’s qualities of ability, benevolence, and integrity, and the perception of these individual qualities is actually the perception of the individual’s trustworthiness (Mayer et al., 1995). Simply stated, individuals who oppose moral violations are considered trustworthy. Trustworthiness is the premise and basis of interpersonal trust (Sun et al., 2023; Wang and Murnighan, 2017), which is composed of both cognitive and emotional trust. The perception of ability and integrity contributes to cognitive-based trust (McAllister, 1995), and the perception of benevolence contributes to emotion-based trust (Dirks and Ferrin, 2002). Therefore, in contrast to neutral judgments, opposed judgments not only directly affect interpersonal trust but also indirectly affect interpersonal trust by affecting trustworthiness. That is, trustworthiness plays a partial mediating role. In the moral violation scenario involved in this study, the intention to oppose moral violation is clear, and the intention to approve moral violation is vague (Carlson et al., 2022). Individuals may actually approve of such moral violations, or they may contrarily approve them for other reasons, such as pressure from peer relationships. Moreover, owing to the low degree of socialization of adolescents during this period, it may not be easy to associate approved judgments with individual moral qualities, so approved judgments do not predict well bystanders’ interpersonal trust. In addition, because the moral violation scenario used in this study was in a school class and the consequences of the violation were relatively light, there was no significant difference in the perceptions of trustworthiness and interpersonal trust of the assessors who made the neutral and approved judgments. Therefore, compared with neutral judgment, trustworthiness plays no mediating role in the influence of approved judgment on interpersonal trust. The costly signaling theory also supports this result, whereas an opposed judgment requires individuals to pay the corresponding cost and bear the risk of retaliation by the violators, which sends a signal to the bystander that the evaluator is trustworthy and increases bystanders’ interpersonal trust. An approved judgment may send a weak signal to bystanders, causing trustworthiness to play no role in the impact of an approved judgment on bystanders’ interpersonal trust (BliegeBird and Smith, 2005; Gintis et al., 2001).

### 4.1 Implications of the study

This study has important theoretical significance and practical value. With respect to theoretical significance, previous studies have focused more on the influence of moral judgment on the evaluator himself or herself. This study expands on this to verify the spillover effect of moral judgment on the interpersonal trust of bystanders. Moreover, this study, which was conducted in the context of an Eastern culture, differs culturally from most studies conducted in Western cultures, and culture is an important factor affecting individual cognition and behavior. Accordingly, this study once again demonstrates the relationship between moral judgment and bystanders’ interpersonal trust from a cross-cultural perspective. In terms of practical value, this study revealed that individual moral judgments directly affect the interpersonal trust of bystanders, which may motivate individuals to make prudent moral judgments in public to win the trust of more bystanders. Furthermore, this study revealed that moral judgment indirectly influences interpersonal trust by influencing bystanders’ perceptions of trustworthiness, which then strengthens the importance of trust building qualities such as ability, benevolence and integrity. These findings contribute to the individual’s understanding of the process of building trust and show how to demonstrate their good qualities through moral judgment in social interactions to improve their reliability and gain the trust of others.

### 4.2 Limitations and future research

Although this study reveals the development mechanism of interpersonal trust during the process of social interaction and the mediating role of trustworthiness, there are several limitations. First, the sample size of this study was not very representative. The sample size was selected based on some criteria, which restricts the sample to a more specific population. Future studies could consider reducing the number of inclusion criteria and generalizing the findings to a broader population. Second, the monitor, who has more rights and responsibilities than do ordinary students, made moral judgments in this study, thus implying that the monitor’s moral judgment had greater authority. In the future, whether there exists such a relationship between the moral judgment of ordinary students and interpersonal trust should be further examined. Third, given that people’s attitudes and behaviors are not always consistent, future research should consider using behavioral paradigms instead of the scales used in this study to measure the behavioral performance of interpersonal trust. Finally, the relationship between moral judgment and interpersonal trust under varying levels of moral violation can be investigated in the future to improve the external validity of such studies, as the degree of moral violation in the scenario used in this study was relatively small, whereas the degrees of moral violations in real life are not equal.

Moreover, this study has important implications for educational practice. In the face of moral violations among students, individuals with influential roles, such as teachers and monitors, should help students make correct moral judgments in a timely manner, which, in turn, improves their (the teachers and monitors) level of trustworthiness and helps them to better manage student behavior. In addition, when providing moral education programs for students, schools should focus on the value of moral knowledge, encourage students to establish correct moral values, and support students as they render moral judgments on moral violations. Taken together, these actions will improve interpersonal trust among students and promote win–win cooperation among young people.

## 5 Conclusion

This study explored the influence of moral judgment on interpersonal trust and its underlying mechanisms via structural equation modeling. Two significant conclusions were reached.(1) Bystanders’ interpersonal trust in the moral evaluator is impacted by the bystanders’ moral judgment of moral transgressions. Compared with people who make neutral and approved judgments, bystanders have greater trust in people who make opposed judgment. (2) Trustworthiness plays a mediating role in the influence of moral judgment on interpersonal trust; that is, moral judgment affects bystanders’ perceptions of trustworthiness and subsequently affects bystanders’ interpersonal trust.

## Data Availability

The raw data supporting the conclusions of this article will be made available by the authors, without undue reservation.
